# Beyond Public Health and Private Choice: Breastfeeding, Embodiment and Public Health Ethics

**DOI:** 10.1007/s41649-023-00259-0

**Published:** 2023-10-26

**Authors:** Supriya Subramani

**Affiliations:** Sydney Health Ethics, School of Public Health, Faculty of Medicine and Health, The University of Sydney, Sydney, Australia

**Keywords:** Breastfeeding intervention, Public health, India, Lived experiences, Embodiment

## Abstract

The key objective of this paper is to emphasize the importance of acknowledging breastfeeding as an embodied social practice within interventions related to breastfeeding and lactation and illustrate how this recognition holds implications for public health ethics debates. Recent scholarship has shown that breastfeeding and lactation support interventions undermine women’s autonomy. However, substantial discourse is required to determine how to align with public health goals while also recognizing the embodied experiences of breastfeeding and lactating individuals. Presently, interventions in this realm predominantly revolve around health-related messaging and the promotion of individual behaviors, often neglecting the systemic and structural factors that influence choices and practices. I closely examine breastfeeding interventions in India, in particular Mothers’ Absolute Affection health promotion program, along with breastfeeding narratives. I argue that for such interventions to evolve, they must acknowledge the intrinsic embodied social nature of breastfeeding during their design and implementation. Furthermore, it is important to emphasize that achieving equity and justice objectives necessitates moving beyond the confines of both conventional public health frameworks and frameworks solely centered on private choices. Instead, a more encompassing approach that embraces the concept of embodiment should be adopted.

## Embracing Breastfeeding Mothers and Lactating Persons

In recent years, numerous studies have demonstrated the many health and social benefits of breastfeeding (Pérez-Escamilla 2020; Sankar et al. 2015; Victora et al. 2016). However, globally, only 41% of infants under 6 months of age are exclusively breastfed (Gupta et al. 2019). At 12 months, breastfeeding prevalence is the highest in South Asia, parts of Africa, and Latin America, while it is lowest in high-income countries, and indicators other than early initiation of breastfeeding decrease with national wealth (Victora et al. 2016). There are a number of factors that contribute to this concern, including social, cultural, political, and economic factors. A number of international and national organizations have proposed effective interventions and strategies in order to advance and promote breastfeeding. WHO/UNICEF’s Baby Friendly Hospital Initiative (BFHI) is an example.

Breastfeeding and lactation interventions have been criticized for failing to consider the experience and knowledge of a woman and lactating persons (Kukla 2006; MacKay 2021; Preston-Roedder et al. 2019; Shaw 2004; Wall 2001; Wolf 2007). Several scholars have criticized breastfeeding promotion, pointing that interventions have failed to take into account the social and material constraints that significantly influence infant feeding decisions (Barnhill and Morain 2015; MacKay 2021; Shaw 2004; Stearns 1999; Taylor and Wallace 2012; Wall 2001; Wolf 2007). Studies illustrate that mothers are viewed as solely responsible for the health of their children and that they are given moral pressure to breastfeed or risk being labelled as “bad mothers” if they do not (Kukla 2006, 2008; Lee 2018; Leeming et al. 2013; Wall 2001). In order for breastfeeding interventions to be effective and ethical, women and lactating persons lived experiences and knowledge must be acknowledged, as it creates an opportunity for politics of motherhood and infant feeding within a larger social, economic, cultural, and political context that prevents lactating persons from breastfeeding. Additionally, taking into consideration the social construction of target populations in public health interventions (Schneider and Ingram 1993), I argue that these interventions construct a certain target population based on assumptions shaped by ideology, politics, and culture. Women who breastfeed are often targeted and constructed as “good mothers” by “good motherhood” ideology (Burns and Schmied 2017; Lock 2015). Moreover, breastfeeding and lactation interventions require an intersectional approach, since gender, class, caste, race, ethnicity, sexual orientation, and any other social identity have an impact on lactating bodies’ experiences within specific social, cultural and political contexts. Taking an intersectional approach to public health interventions such as breastfeeding interventions along with acknowledging the lived experiences of women and lactating persons allows us to critically examine the dominant choice paradigm.

In general, breastfeeding interventions can be categorized into three groups: protection, which limits the sale of infant formula; promotion, which educates and informs individuals and communities about breastfeeding’s benefits; and support, which reduces the social, economic, cultural, and political barriers women face when initiating and maintaining breastfeeding. There is a great deal of criticism focusing only on protection, prevention, and promotion interventions, without engaging much with support interventions (Smith 2018). Unless the social, cultural, economic, and political conditions of breastfeeding are addressed, desired breastfeeding rates become impossible and exclude the experiences of lactating individuals. One needs to acknowledge that breastfeeding decisions happens within the backdrop of one’s cultural context (Hays 1996). Breastfeeding interventions that target only individual behavior and make women and lactating persons feel shame and guilt have been criticized by several scholars recently (Dowling et al. 2018; Leeming 2018; Taylor and Wallace 2012; Thomson et al. 2015). Recently, a Lancet series article emphasized the importance of political economy research that examines structural factors contributing to a lack of breastfeeding support in healthcare systems and, as part of the closing the breastfeeding rate gap, these authors stressed the importance of breastfeeding being regarded as care work (Pérez-Escamilla et al. 2023). As long as supportive factors are not acknowledged and incorporated, breastfeeding would become a moralizing practice perpetuating the “good mother” narrative (Lee 2018), and thus non-structural approach adopts “framework of blame” especially to mothers (Sridhar 2010).

I briefly discuss in the next section the importance of valuing the embodied lived experiences of breastfeeding women and lactating persons within the context of breastfeeding and lactation interventions. In India, women and lactating persons cite a lack of support systems as a reason for their inability to breastfeed (Jacob 2018; Sridhar 2010; Van Hollen 2003; Van Hollen 2011). So, in the “Missing Maternal Experiences: The Indian Context” section, I will examine how moralizing discourses about women and motherhood function within an Indian context and critically evaluate national breastfeeding program—Mother Absolute Affection. In the “Breastfeeding, Embodiment and Public Health Ethics” section, I present normative justifications for acknowledging breastfeeding as an embodied social practice. This recognition has the potential to reshape the perception surrounding breastfeeding advocacy, promotion, and support. These efforts can then effectively contribute to broader public health objectives concerning maternal and infant well-being, transcending the dichotomized understanding of public health and personal choice.

## Embodied Breastfeeding: Respecting Women and Lactating Person’s Lived Experiences

The concept of embodiment helps us to overcome the dualistic separation of mind/body or self/other and emphasizes how one’s body is central to being in the world, and how it shapes one’s subjectivity (understanding of oneself and others) and relationship to the world and with others. In other words, embodiment refers to the concept of our experiences, perceptions, emotions, and actions being deeply entwined with our physical bodies (Merleau-Ponty 2004). Informed by feminist scholars (Scully 2014; Fineman 2004; Young 2020), phenomenologists (Dolezal 2015; Svenaeus 2017; Lee 2018; Merleau-Ponty 2004), and political philosopher (Sandel 1998), I see breastfeeding as an embodied social practice that unsettles the notion of subjectivity and demands us to rethink the notion of liberal conception of individual. Breastfeeding as a dyadic act inherently involves caring for other/child but at the same time, it makes one bodily practice self-conscious within a particular context. As Hausman (2004) mentions “As a practice, breastfeeding is a daily pattern of embodied living; support for it is a recognition of the reproductive burden women experience through their bodies. p. 276.” The practice of breastfeeding transcends the public-private divide; it is an intersubjective experience. An understanding of breastfeeding based on social position and socioeconomic and cultural context situates mothers and lactating individuals in a relationship to their bodies. The feminist literature sheds light on the larger patriarchal as well as capitalist mechanisms that oppress women and lactating persons and encourage breastfeeding to be viewed as an act of care and empowerment (Binns and Lee 2019; Lee 2018; Leeming et al. 2013). Understanding this literature and the different perspectives requires a close examination of the lived experiences of breastfeeding women within the contexts in which they find themselves.

In some circles, breastfeeding is considered natural and morally correct. Although many scholars question the idea of “natural” and ask us to reflect on it (Martucci and Barnhill 2018), breastfeeding is still considered natural, causing many women to perceive themselves as “unnatural” or “bad mothers” when they cannot or decide not to breastfeed. Several feminist scholars have argued against this. Surrogacy and IVF, for example, challenge biological and natural conceptions of motherhood. Ideology is another significant constraint to breastfeeding. Ideology shapes women’s experiences of their bodies, making them feel like “good mothers or bad mothers.” The low supply of milk is perceived by women as a bodily failing, for example. Mothers’ evaluation of their role, fitness, and values are also influenced by recurring images of a “good mother” and “pure breastmilk” in interventions, policy, and legal discourse. Consequently, addressing dominant ideologies of motherhood and taking into consideration social, economic, and cultural barriers helps realize the right to breastfeed, respect maternal experience, and achieve public health goals.

Often, public health advocates overemphasize medical evidence without addressing and incorporating the experiences of women and lactating persons in particular context. There has been a great deal of literature that demonstrates the larger structural barriers to breastfeeding, for example the lack of support system when returning to work or the difficulties of breastfeeding in public. There is evidence from a number of ethnographic studies that breastfeeding causes women to feel shame, guilt, and anger (Burton et al. 2022; Dowling et al. 2018; Leeming 2018; Taylor and Wallace 2012; Thomson et al. 2015; Tomori et al. 2016). Therefore, breastfeeding experiences must be respected as part of the interventions, going beyond the choice framework and considering sociocultural and economic barriers. Recent studies show that most breastfeeding and lactation interventions are educational, focusing on behavior and action of individuals, rather than environmental or support-based, and they focus primarily on changing individual behavior especially breastfeeding mother or immediate family (Balogun et al. 2016; Khatib et al. 2023; Sinha et al. 2015). Health messaging and counseling, for example.

According to Buchanan (2019), public health interventions can be divided into educational and environmental interventions. The goal of educational interventions is to change the knowledge and beliefs of individuals or populations, and the goal of environmental interventions is to tackle the social determinants of health. Numerous randomized controlled trials have been conducted on various breastfeeding interventions in an effort to enhance breastfeeding practices. Despite these efforts, the global breastfeeding rate has remained relatively stable at around 40% since 1990. Although it is widely acknowledged that breastfeeding is influenced by a person’s lived experience, sociocultural economic context, and biology (Van Esterik 2012), many global health and public health interventions tend to focus on outcomes and determinants dominantly through educational interventions without much attention to larger systemic barriers. Furthermore, scholars point out that these interventions focus on the product, namely human milk, which is decontextualized, medicalized, and disembodied (Giles 2012; Lee 2018). The “one size fits all” approach or lumping all lactating women and bodies together would create a moral space based on how their goals align with public health objectives. In focusing only on disembodied agency or choice paradigm, these interventions ignore how women and breastfeeding practices are embedded in larger sociocultural structures. In later “Embracing breastfeeding mothers and lactating persons” section argues that it becomes necessary to take embodiment seriously in public health ethics discourse in order to achieve equity and social justice goals when planning and implementing interventions.

In the following section, I will outline the underlying ideology of Indian breastfeeding interventions. I will illustrate how the promotion effort adopts a choice framework focusing on mothers and their responsibilities as natural and instinctive as they ensure the proper development of their children through Mothers’ Absolute Affection program. Furthermore, by contextualizing the program within the Indian socio-economic, cultural, and political context, I will show it overlooks crucial social, cultural and material obstacles to breastfeeding, and treats breastfeeding as dominantly a mother’s responsibility.

## Missing Maternal Experiences: The Indian Context

(Breastmilk is nature’s gift) 

(Mother’s milk is nectar) 

*Breastfeeding and Breastmilk promotional slogans*

*Department of Health and Family Welfare Services, Government of Karnataka*

It is similar to the early European ideal of natural motherhood and breastfeeding as a womanly duty that was valorized by early European scholars (Golden 1996), where identity of motherhood and mothering were important socialisation processes for Indian women, and regarded as natural. In much of Indian literature and mythology, mother is deified, and mother is a metaphor for nation and language (Krishnaraj 2010; Mitra 2020). In these discussions, the mother’s natural role is to nurture her child, especially a male child. As a reflection on this archetype of the mother, some scholars have critically reflected on how women are viewed as “objects” and breastfeeding becomes a form of labour (Mitra 2020). Breastfeeding is heavily moralized, as we have seen in feminist academic debates in the US, Canada, and UK, as being a “natural and maternal duty” (Shaw 2004; Stearns 1999; Wall 2001; Wolf 2007). While recently, it is much admiring to see a passionate push to breastfeeding to transform maternal and childcare (Jindal 2020), especially in rural India, the larger cultural context and the embodied experience of women coming from diverse settings is yet to be explored critically.

There are regional differences between breastfeeding practices in India, with the highest prevalence of Exclusive Breastfeeding (EBF), 79.2% at 0–4 months, occurring in the southern region and the lowest prevalence (68%) occurring in the north-eastern region (Ogbo et al. 2019), which is much above the global breastfeeding rate. A significant finding is that exclusive breastfeeding in the south fell faster at 5 months, to 43.7%, than in the north-east (54%). In addition, exclusive breastfeeding and continued breastfeeding rates in urban areas are lower than in socio-economically depressed eastern and north-eastern areas of the country (Ogbo et al. 2019). It has been demonstrated in the global breastfeeding literature that higher income and education are associated with better breastfeeding practices (Birhan et al. 2022; Holowko et al. 2016; Standish and Parker 2022). It should be noted that in India, the relationship between education and breastfeeding is complex. In the southern region of India, higher maternal education was associated with low breastfeeding, whereas in the central region of India, higher maternal education was associated with exclusive breastfeeding (Ogbo et al. 2019). This demands us to look critically at existing breastfeeding interventions at national and state levels. According to some studies, there are several reasons for this difference, including obesity among urban women, lack of breastfeeding opportunities, the ability to arrange infant formula economically, increased work involvement, inflexible schedules, and limited childcare leave and concerns regarding privacy or safety (Bhanderi et al. 2019; Ogbo et al. 2019; Ramani et al. 2019). Furthermore, Jacob (2018) highlights through Sridhar’s work on the Tamil Nadu Integrated Nutrition Project that the underlying assumptions of interventions and programs do not accommodate structural inequalities and limitations and are based upon narrow biomedical and health economic models. As an example, many women and respondents do not breastfeed due to ignorance, but because of poverty, domestic violence, lack of financial autonomy or the existing labour system (Shroff et al. 2011; Zureick-Brown et al. 2013). While these studies provide important insights into the experience of breastfeeding and lactating individuals, there is a lack of in-depth qualitative research on the lived experiences of lactating persons within the Indian context when it comes to nutrition and breastfeeding research.

Maithreyi Krishnaraj (2010) edited “Motherhood in India: Glorification without Empowerment?” which explores the ideology of motherhood in the context of Indian women’s experiences as mothers. There is much evidence in this work that women’s reproductive biology, particularly their ability to bear and nourish children through breastfeeding, has influenced not only the status of women within their families and kin groups but also the policy and program of the government (Krishnaraj 2010). In literary texts, motherhood is often depicted as a site of sanctity, purity, and divinity. The Tamil literature, for instance, depicts mothers as possessing certain mystical qualities that enable them to produce “the milk of valour” for their sons. Hence, they are made warriors by infusing their blood with bravery and courage. Present-day rituals and everyday practices surrounding pregnancy and mothering revolve around diet, feeding children, movement, place of delivery—all matters that affect the entire family, not just the mother. Experiences of pregnant women and breastfeeding reveal the complex social-cultural context of this process. This is because these practices are embedded in a larger set of cultural norms and values. For example, the type of food a pregnant woman is expected to eat is determined by the culture she is a part of, as is the way she is expected to move and the place where she will give birth. All of these practices are shaped by the culture in which she lives and her family’s expectations, and this influences her breastfeeding practices. The following excerpt from an ethnography from Malad (suburb of Mumbai) by Pandey (2010) best illustrates this point.

Nirmala is a mother of four daughters and two sons. In her caste, the daughter-in-law does not go for the first delivery to her natal home. Nirmala did not observe the caste rules and brought ‘bad luck’ to the family by delivering two daughters. Subsequently, the later two deliveries were handled by the mother-in-law who was very superstitious. The last delivery dealt a blow to Nirmala’s health. No one seems to be much concerned about Nirmala’s constant health problems after the last delivery. She has a constant problem of body ache and weakness. ‘I had to breastfeed all my children till fifteen months. My mother-in-law feels that breast milk increases children’s self-confidence, but this has affected my health,’ she complained. (Pandey 2010, 306)

In India, like elsewhere, breastfeeding in public and the idea of breastfeeding among women and the general public raise important questions regarding women’s bodies, sexuality, and parenthood. It becomes a contested space when it comes to the public and private perception of women’s bodies when breastfeeding. Recently, a number of incidents have been reported involving breastfeeding in public places. In south Kolkata (Mahara 2018), a young woman wrote about her terrible experience on Facebook and the mall authorities responded as follows: “Funny you found this to be an issue because breastfeeding is not allowed on the floor for a number of reasons… please make sure you do your home chores at home and not in the mall … It's not like your baby needs to be breastfed at any moment so you need arrangements to be made for you at any public area to breastfeed your child anywhere you wish to … we cannot compromise the privacy of other people in public places can we?”

Breastfeeding leaves contradictory messages regarding women’s breasts and breastfeeding as can be seen from the above excerpt and women’s experiences captured in social media and the news. The concept of privacy is bizarrely understood, and breastfeeding is viewed as a domestic chore that can only be done in a private room or home. It is important to note that the male and sexual gaze on the breast, and responses such as “indecent” and “domestic chore,” private and accommodating others’ perceived comforts restrict women from expressing their right to breastfeed and act as a significant barrier to breastfeeding. It is particularly challenging for urban working women, especially in the informal economy, who do not have the opportunity to breastfeed. In one sense, the act and the breast milk are considered natural, pure, and a sign of maternal love, while in the other sense, they are regarded as impure, indecent, and sexual. It is important to note that when breastfeeding in public is not socially and culturally acceptable, it becomes a vulnerable act that can be distressing for a woman, thus negatively affecting her decision to continue nursing. Breastfeeding women and lactating persons experience their surroundings very differently, as the boundaries between private and public spaces blur. For example, breastfeeding women must negotiate their space and their bodies in the process of breastfeeding. Therefore, identity, power, and social structures have a significant influence on how women negotiate their bodies and make decisions, and thus impact their capacity to exercise their decisions. Generally, it has been difficult for women to occupy public space in India (Phadke et al. 2011; Roy and Bailey 2021), and even those predominant spaces, such as malls, parks, public transportation, and bus stops, are difficult for women to breastfeed since these spaces are experienced differently at various times by women from diverse socio-economic backgrounds.

An online survey conducted by Momspresso (Dhoop 2019), investigated the primary challenges faced by breastfeeding Indian mothers. While most of the participants who did the survey were middle and upper class, the main concerns were social stigmas, particularly perceived as “shameful, disgrace, indecent or embarrassing” as society views the female breast as an object of sexual desire, and the lack of clean and hygienic nursing facilities. As indicated in the survey media (Momspresso 2019) and in several incidents captured by, breastfeeding is perceived as a feminine, private, and domestic activity, which makes it difficult for women to access public spaces and limits their autonomy. Therefore, it becomes necessary to gain a deeper understanding of the experiences and challenges women face within a particular social and cultural context. The need to critically examine how women perceive their bodies and the act of breastfeeding across different socio-economic groups becomes imperative, as well as the challenges they face. While formal female labour participation in India has declined from 32% in 2005 to 19% in 2021 (International Labour Organization 2023), many women are employed in the informal sector and are not documented. In the informal sector, most working women are exposed to exploitation as a result of a lack of formal protections, such as maternity benefits, child care access, and employment protections (Horwood et al. 2020).

Several programs have been implemented by the Indian government to improve maternal and child health, including the Pradhan Mantri Surakshit Matritva Abhiyan (PMSMA), the Pradhan Mantri Matru Vandana Yojana (PMMVY), the Maternity Leave Incentive Scheme, and the Pan-India Maternity Benefit Program (Kiran et al. 2022). In spite of its strong commitment to infant and young child feeding, India only scored moderately in terms of policies, programs, and practices. As a result of these initiatives, India launched the Mothers’ Absolute Affection (MAA) program (2016) in order to enhance the skills and capacities of health care professionals so as to enhance their ability to promote, protect, and support women and lactating mothers. The Indian national breastfeeding program “Mothers’ Absolute Affection,” as well as various state government posters, flyers, and advertisements and private organizations aimed at promoting breastfeeding, primarily target individual women’s behaviors and choices. These educational interventions, along with the larger moral climates of motherhood in patriarchal society, suggest that breastfeeding and human milk are absolute responsibilities of mothers and lactating bodies and are intertwined with the concept of mothers’ love and affection. The conception of breastfeeding as a symbol of infinite love and selflessness has been criticized by a number of scholars. Guenther (2006) argues that infinite responsibility confirms the patriarchal ideal of the self-sacrificing woman, and the moral motherhood associated with breastfeeding. Breastfeeding is not an ethical necessity or an obligation necessary to maintain woman’s identity or to be a good mother. The idea of breastfeeding as an embodied social practice allows us to open up a dialogue and challenge the notion of “good mother,” “good woman,” or “maternal body” within a given context and reflect on the larger socio-economic, ideological, and political factors that impact lactating individuals’ choices and decisions. Recently, maternal subjectivity is examined within breastfeeding context. Lee (2018), have delved deeply into the ethics of subjectivity and the construction of maternal subjectivity. Lee draws inspiration from Levinas proposing that breastfeeding serves as a response to hunger and embodies a sense of responsibility towards the other. Moreover, Lee’s exploration of subjectivity is rooted in the ethical and poetic dimensions of breastfeeding. The purpose of this article is not to go in detail about this conceptual and theoretical work, but rather to argue that breastfeeding should be considered as an embodied social practice within breastfeeding interventions and illustrate why it matters to public health ethics debates.

In the Indian context, breastfeeding literature typically focuses on capturing breastfeeding rates, as well as identifying the key determinants and barriers to achieve public health objectives and some of them engage in critical analysis of policy (Bhanderi et al. 2019; Nishimura et al. 2018; Ogbo et al. 2019; Ramani et al. 2019; Senanayake et al. 2019; Sethi and Murira 2023). Although these studies are essential to understanding breastfeeding barriers and macro level factors, qualitative and phenomenological research is lacking in order to understand the maternal and breastfeeding experiences, how these experiences are situated within a larger socio-cultural and ideological context, and how they influence breastfeeding practices. This points to the need for more qualitative research in this area. Due to natural health movements and motherhood movements against the formula industry and medicalization of infant feeding in North America and Europe (Martucci and Barnhill 2018), Indian promotional campaigns often resemble similar narrative. Blum’s (1993) works provide insight into how maternalist and medical models construct a moral image of mothers and their choices. According to a medical model, the goal is to increase the nutrition and development of the child by providing awareness and support. In the maternalist model, it valorizes self-sacrifice, nurturing, caring mother and its benefit for child. Both of these models place little emphasis on the well-being of women and lactating persons, and their experiences and agency, and are primarily concerned with optimizing children’s health. In these models, the term “natural and maternal instinct” is used. This has moral implications for mothers and lactating persons as this implies a certain role and responsibility for being a “good mother.” This can be seen in the Indian promotional campaign slogans and interventions that primarily focus on the choices and behaviors of women. This individualistic approach has been demonstrated to be ineffective as well as unstable by a number of scholars (Brubaker and Dillaway 2008, 2009). A primary concern with employing these models in breastfeeding interventions is that they do not take into account the lived realities of women and lactating individuals, nor do they acknowledge the fact that breastfeeding is a socially embodied and gendered practice within a particular social-cultural ideological context. As a result, these interventions view breastfeeding as a responsibility and duty of mothers without considering the larger social and material support systems that lactating persons require to practice breastfeeding.

## Breastfeeding, Embodiment and Public Health Ethics

Based on understanding of practice and embodiment literature (Csordas 1990; Desjarlais and Jason Throop 2011; Lee 2018; Shaw 2004; Stearns 2013), one can understand breastfeeding as a gendered and socially embodied practice. These works explore embodiment, corporality, the lived experience and its relationship to self and others. In the previous sections, we saw that the current dominant breastfeeding interventions consider breastfeeding to be a “somatic practice” and as a “morally insignificant” practice that can be overtly “moralized” by adopting a maternalist or medical approach. Breastfeeding as an act is ethical, as Shaw (2004) argues “it is erroneous to view lactation simply as a natural and passive somatic act that is morally or ethically vacuous. Since lactation is embedded historically in socio-cultural practices, it falls squarely within the purview of ethics” (p. 101). Through acknowledging breastfeeding as an embodied social practice (Stearns 1999, 2013) and women’s lived experiences with breastfeeding and lactation interventions, one can address the challenges women and lactating persons navigate in particular social and moral contexts. It is only by understanding their lived experiences and embodied emotions, such as anger, guilt, and shame, and experiences that we can determine why these interventions fail or are ineffective. Embracing lived experiences within a socio-ecological model (Schölmerich and Kawachi 2016) is essential for moving beyond public health and private choice as well as critically evaluating how interventions in public health, such as breastfeeding and lactation interventions, construct particular women and lactating bodies.

It is widely accepted that health is a social phenomenon in public health philosophy and public health ethics. It has also been central to public health thinking as it has moved away from individualistic behavior and resisted the biomedical model of health. In light of the importance of addressing substantive and procedural issues in public health, feminist philosophy and critical scholarship has influenced researchers in the bioethics field to focus on gender discrimination and structural inequality and to emphasize the interconnectedness of body and mind with subjectivity. Despite the increasing recognition of intersectionality and the focus on the lived experiences of marginalized groups in public health (ethics), especially within feminist scholarship (Rogers et al. 2022; Rogers 2006), it is also significant to emphasize why embodiment is crucial to public health ethics, especially when implementing interventions (including breastfeeding interventions) to improve the health and well-being of populations. In order to achieve a just society, we must engage in public health ethics conversation that is based on a version of a public philosophy that includes individuals’ embodied vulnerability. Because the way one navigates the world is influenced by the embodied body, especially marginalized bodies (hooks 2014; Young 2020). Given the scope of this paper, I will focus on two key normative reasons why public health ethics should value embodiment.

As a first reason, embodiment matters because they shape one’s experiences in a particular social context and may have an impact on one’s ability to exercise autonomy. Here, autonomy is the action or decision that considers both the mind and body, reason and emotion, not as dichotomous concepts but as an intricate interplay between our physical embodied social body, perception, emotions, and behavior. To achieve just and equitable society, which is one of the key values of public health ethics, it is necessary to acknowledge how social institutions within systemic inequalities affect the lived experience of an individual. Depending on an individual’s physical and environmental capabilities, embodiment and lived experiences may limit or enhance one’s opportunities. The reality of breastfeeding in different contexts within different breastfeeding and lactating communities is, however, much more complex, as it involves a process of relearning and learning to navigate once familiar everyday practices. If a lactating individual feels stigmatized by public breastfeeding, as I have demonstrated in previous sections, they may be discouraged from doing so. Additionally, the way in which women and transpersons are socialized in patriarchal societies may have an impact on the decisions they make regarding breastfeeding and the techniques they use. For this reason, public health interventions must examine how lactating persons experience their embodiment, so as to account for how one’s (maternal) autonomy may be compromised or diminished. In the absence of first-person accounts that illustrate the lived, embodied social practice of breastfeeding, especially from the perspective of autonomy, it is easy to assume breastfeeding will unproblematically promote maternal and child health. For instance, one cannot fully understand the challenges faced by urban working women when breastfeeding unless one investigates the difficulties they encounter in the informal sector, especially since this interferes with their livelihoods.

As a second normative reason, embodiment plays an important role in public health ethics because of its ability to illustrate the magnitude of harm/burden and unfairness. Particularly, since it emphasizes the disparities that result from the social determinants of health (Krieger 2001). Since one of the fundamental values of public health ethics is to influence access and overall well-being, addressing these disparities by acknowledging their root causes and advocating for policies that promote health equity are essential. Breastfeeding mothers and lactating individuals experience harm or suffering as a result of breastfeeding, whether it is physical pain or psychological suffering, such as stigma, guilt or shame or due to situation social, environment, political and economic context. Because breastfeeding is an embodied social practice, certain activities may feel burdensome or painful, even if they are temporary. To meet this need, society at large, and particularly public institutions, should provide support systems for breastfeeding without relying on individualistic choice models. For example, it is unfair to expect women working in the informal sector to drop out of the workforce to breastfeed their children. Also, a recent study revealed that women were better prepared and equipped to handle breastfeeding pressure if they knew breastfeeding problems and challenges (Cato et al. 2020). As burdens and suffering are experiential, studying how lactating individuals experience their embodiment may help us understand the magnitude of suffering people experience. Therefore, a better understanding of the nature and magnitude of the suffering and burden will require examining people’s first-person experiences of embodiment. In addition, examining public health interventions through the lens of suffering aids in mitigating or alleviating its adverse effects on breastfeeding mothers. Furthermore, given that equity and justice are key principles of public health ethics, it is essential to acknowledge that breastfeeding is an embodied social practice in order to accomplish practice these values in the design and implementation of breastfeeding interventions. Given the increasing recognition of the underlying normative framework of public health ethics based on complex systems (Wilson 2009), life course and relatedness approach (Jones et al. 2019), it becomes critical to emphasize social body, embodiment and lived experiences within public health discourse. Infant mortality, for example, is understood not only in terms of immutable biological and meteorological factors but also in terms of motherhood, nutrition, and social class which are also influenced by cultural context. By focusing on embodied subjectivity, we can position the lactating person’s needs to the forefront and provide structural opportunities to exercise one’s capacities and breastfeeding decisions without falling into the trap of agency vs structure or individual health vs population health tired trap.

Recently, studies emphasize the importance of combining educational interventions and community-based interventions in promoting breastfeeding and child nutrition (Haroon et al. 2013; Khatib et al. 2023; Sinha et al. 2015). However, to improve breastfeeding and child nutrition, it is important to maximize the support system beyond the women and family. It is necessary to critically analyze how one can achieve equity and justice for breastfeeding women and lactating persons in India based on the larger cultural barriers of a lack of public breastfeeding support, institutional support to assist breastfeeding persons, and the preference for breastfeeding male children and those with a higher birth order. There is a long history in which wet nursing has been associated with slavery in the USA and elsewhere. In India, Dalit women and mothers who are from southern India have been forced to become wet nurses (Uppuleti 2023). In Breast Stories by Mahasweta Devi, translated by Gayatri Chakravorty Spivak (Devi and Spivak 2006), breast becomes a symbol of social exploitation where women’s identity and body becomes “object”. Lee (Lee 2018, 2019) critically engage on the nature of human milk exchange and its implications within existing exploitative systems. These discussions demands us to examine critically how caste, class, and existing social hierarchical dynamics play out in the contemporary commercialization of human milk banks within the Indian context. Furthermore, as we saw in earlier sections in India different barriers and determinants exist for different groups of women in different regions, and that interventions should not be targeted based on a one-size-fits-all approach.

While there has been increased implementation of a number of interventions in India, such as the Integrated Child Development Services and Mothers’ Absolute Affection, no clear studies have been conducted to establish that these interventions have resulted in an increase in continuing breastfeeding behavior. Many quantitative studies have been conducted to analyze breastfeeding barriers and their determinants. However, contextualizing these data is critical to understanding systemic and structural barriers. Public health scholars have been advocating for focusing on collective societal approaches that consider gender inequities and socioeconomic inequalities, as well as recognizing breastfeeding as care work, rather than on women’s responsibility to breastfeed (Baker et al. 2023; Cecília Tomori et al. 2022). The shift to resist a market-driven industry cannot be achieved so far as we fail to acknowledge and respect the lived experiences and embodied practices of breastfeeding among lactating individuals, as well as systemic constraints that limit breastfeeding opportunities. This paper thus concludes that taking embodiment seriously not only enhances our understanding of ethical and political theories, but also requires us to make significant changes in how we think about moral and political issues, such as breastfeeding and not just see this as a public health concern alone. Taking into account, the complex social dynamics of breastfeeding can help to ensure that breastfeeding and lactating persons are respected while promoting, protecting, and supporting public health. Therefore, breastfeeding interventions should be understood beyond public health and private choice frameworks by acknowledging the embodied social practice of breastfeeding to achieve equity and social justice.
